# Evaluation of subscapularis tendon tears of the anterosuperior aspect using radial-sequence magnetic resonance imaging

**DOI:** 10.1016/j.jseint.2021.08.005

**Published:** 2021-09-28

**Authors:** Ryosuke Matsushita, Shin Yokoya, Hiroshi Negi, Norimasa Matsubara, Yuji Akiyama, Nobuo Adachi

**Affiliations:** a Department of Orthopaedic Surgery, Graduate School of Biomedical and Health Sciences, Hiroshima University, Hiroshima, Japan; b Department of Clinical Radiology, Hiroshima University Hospital, Hiroshima, Japan

**Keywords:** Rotator cuff, Arthroscopic rotator cuff repair, Subscapularis tendon, Radial magnetic resonance imaging, Classification, Conventional magnetic resonance imaging

## Abstract

**Background:**

Magnetic resonance imaging (MRI) is widely used to diagnose subscapularis tendon tears; however, it is difficult to assess the anterosuperior aspect of these tears. Radial-sequence MRI can reveal the fiber components of the anterosuperior aspect, from perpendicular, by overcoming the partial volume effect. We aimed to classify the insertion of subscapularis tendon tears on radial-sequence MRI and determine the effectiveness of radial-sequence MRI for subscapularis tendon tear assessments.

**Methods:**

We retrospectively investigated 196 patients (mean age, 66.7 ± 9.0 years; 118 men, 78 women) who underwent 1.5 T MRI before arthroscopic rotator cuff repair. Radial-sequence MRI findings of the anterosuperior aspect insertion of the subscapularis tendon were classified into five grades, and intraoperative findings compared with preoperative conventional MRI and radial-sequence MRI. We calculated sensitivity, specificity, accuracy, and positive and negative predictive values. Interobserver and intraobserver reliability for radial-sequence MRI classification was calculated using kappa (κ).

**Results:**

Conventional MRI sensitivity of subscapularis tendon tears was 45.3%; specificity, 95.8%; accuracy, 82.1%; positive predictive value, 80.0%; and negative predictive value, 82.5%. Radial-sequence MRI sensitivity was 92.5%; specificity, 88.1%; accuracy, 89.3%; positive predictive value, 74.2%; and negative predictive value, 96.9%. Sensitivity (*P* < .001), accuracy (*P* = .04), specificity (*P* = .02), and negative predictive values (*P* < .001) in radial-sequence MRI were significantly higher than those in conventional MRI. Intraobserver and interobserver reliabilities for radial-sequence MRI classification were κ = 0.78 and 0.65, respectively, corresponding to high reproducibility, and defined as good.

**Conclusion:**

We provide evidence that radial-sequence MRI is an effective tool to evaluate subscapularis tendon tears, especially before surgery.

The rotator cuff is composed of four tendons of the supraspinatus, infraspinatus, subscapularis, and teres minor muscles and is attached around the humeral head. Among them, the subscapularis tendon (SSC) attaches to the lesser tubercle of the humeral head and contributes to anterior stability. The long head tendon of biceps (LHB) passes through the bicipital groove just lateral to the SSC attachment area, and the condition of the insertion of the SSC relies on the stability of the LHB.^3^

Damage to the rotator cuff causes shoulder pain and dysfunction, often requiring surgery. A subscapularis tendon tear (STT) is thought to cause a decrease in internal rotator muscle strength and increase anterior shoulder joint pain and instability.^25^ It has been commonly reported that most STTs start from the anterosuperior aspect.^27^^,^^30^ Although an STT, which used to be called a hidden lesion, was not regarded as a major clinical problem,^19^^,^^31^ it has been reported that it is associated with approximately 30% of rotator cuff tears (RCTs).^5^ In addition, Nakamura et al^23^ reported that the pain of RCTs with an STT is difficult to improve. Therefore, when a surgeon diagnoses RCTs with an STT, it is important to include the assessment of the anterosuperior aspect tear.

Currently, horizontal, oblique sagittal, and oblique coronal magnetic resonance imaging (MRI) are commonly used to diagnose RCTs. However, the sensitivity of STT diagnosis using conventional MRI (cMRI) is not particularly high.^1^^,^^2^^,^^7^^,^^20^^,^^21^ In cMRI, it is difficult to evaluate the anterosuperior aspect of the SSC because the diagonal view of the SSC attachment area becomes unclear owing to the partial volume effect from tangential projection.^16^

On the other hand, Kubo et al^16^ reported that radial-sequence MRI (rMRI) could be a useful technique for evaluating the condition of the acetabular labrum. rMRI may be a more powerful diagnostic tool than cMRI to accurately diagnose anterosuperior aspect tears. The rotator cuff tendons are also attached to the humerus head in a circumferential shape, similar to the acetabular labrum. Because rMRI draws radially from the center of the humeral head, any part of the rotator cuff tendons can be described vertically. rMRI can reveal the fiber components of the anterosuperior aspect from perpendicular by overcoming the partial volume effect, which can be particularly noticeable with cMRI.

Furukawa et al^8^ investigated 55 patients for diagnostic accuracy of STT using 3.0 T rMRI and reported good results. However, that study had a small number of subjects and used 3.0 T MRI. There is no study of rMRI using only 1.5 T MRI. 3.0 T MRI is superior to 1.5 T MRI in terms of diagnostic performance; however, it is more expensive and is not popular.^4^^,^^11^ It may also be difficult to diagnose STT if the user is unfamiliar with rMRI, and no classification for the insertion of STT on rMRI has been developed.

The purpose of this study was to classify the insertion of STT on rMRI and to determine the effectiveness of using rMRI for the assessment of STT by evaluating each result based on the following: sensitivity, specificity, accuracy, positive predictive value (PPV), and negative predictive value (NPV). We compared the accuracy of the evaluation using rMRI and cMRI. Our hypotheses were that our rMRI classification would be of use to diagnose tears at the insertion of the subscapularis tendon and that rMRI may obtain better results than cMRI using 1.5 T MRI.

## Materials and methods

This diagnostic reliability study was reviewed and approved by the Ethical Committee of our institution.

### Patients

This study retrospectively investigated 196 shoulders that had undergone cMRI and rMRI before arthroscopic rotator cuff repair for RCT from June, 2012 to December, 2018. The mean age was 66.7 ± 9.0 years (range, 34-86 years). A total of 118 patients were male and 78 were female, and there were 130 right shoulders and 66 left shoulders. The exclusion criteria were previous shoulder surgery, calcific tendinitis, and fracture of the lesser tubercle of the humerus.

### MRI protocol

All MRIs were performed in the supine position, with the shoulder slightly externally rotated using a shoulder coil. We used the Ingenia 1.5 T MR system (Philips, North America LCC, Andover, MA, USA). Both cMRI and rMRI images were acquired in one session using the same MRI system for all images of each patient.

The axial view was obtained using a T2-weighted sequence (reception time = 4000 ms; echo time = 100 ms; echo train length = 22; matrix = 304 × 217; slice thickness = 4.5 mm without gap; total scan duration = 2 min). All continuous slices were taken perpendicular to the glenoid surface of the reference coronal images, which were taken preliminarily. Oblique coronal images were also acquired using T2-weighted images (reception time = 4000 ms; echo time = 100 ms; echo train length = 22; matrix = 320 × 230; slice thickness = 4.5 mm without gap; total scan duration = 2.24 min). Continuous slices were taken perpendicular to the scapular spine on an axial view. Oblique sagittal images were acquired using T2-weighted images (reception time = 4000 ms; echo time = 100 ms; echo train length = 24; matrix = 320 × 230; slice thickness = 4.5 mm without gap; total scan duration = 2.8 min). Oblique slices were taken perpendicular to the oblique coronal images.

**Figure 1:**
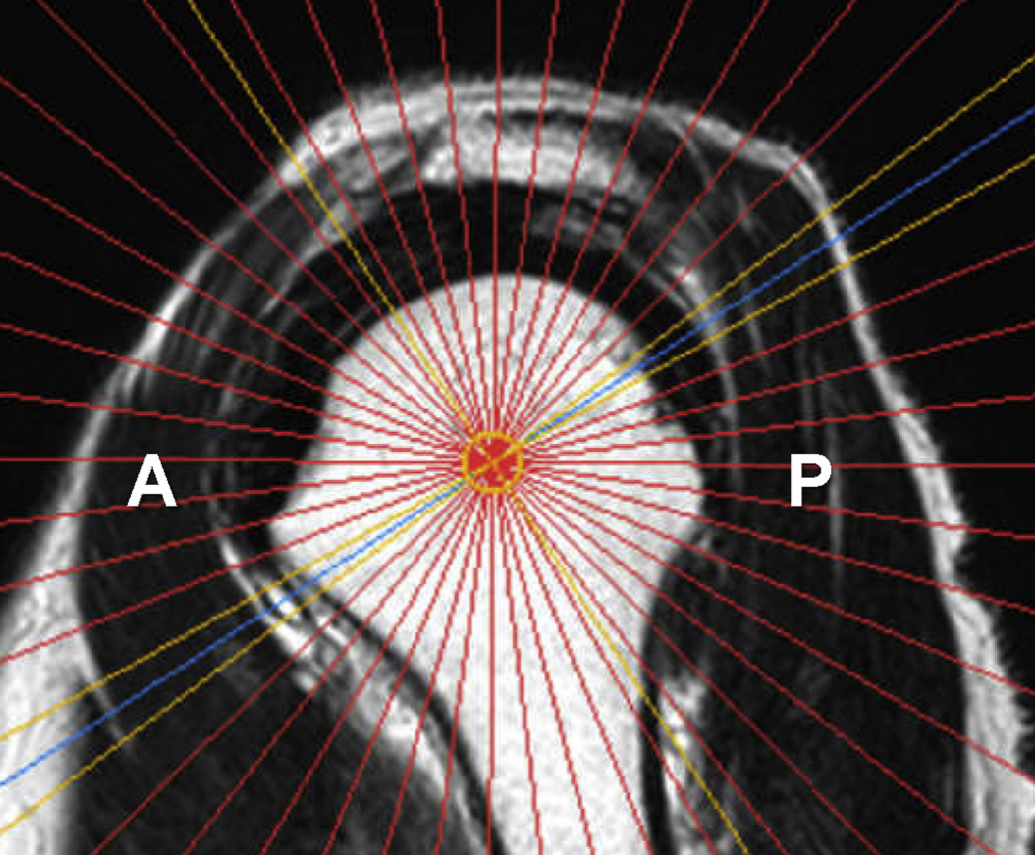
Radial image setting. The center of the humeral head was determined using the sagittal and coronal images. All slices were taken based on the sagittal images, perpendicular to the glenoid surface, and passing through the center of the humeral head, with 24 slices at 7.5° intervals. *A*, anterior; *P*, posterior.

The rMRI images were acquired using T2-weighted images (reception time = 4000 ms; echo time = 100 ms; echo train length = 22; matrix = 352 × 246; slice thickness = 4.5 mm without gap; total scan duration = 2.56 min). The center of the humeral head was determined using the sagittal and coronal images. All slices were taken based on the sagittal images, perpendicular to the glenoid surface, and passing through the center of the humeral head, with 24 slices at 7.5° intervals (Fig. 1). Filming this radial-sequence image took approximately 5 min.

### MRI evaluation

**Figure 2:**
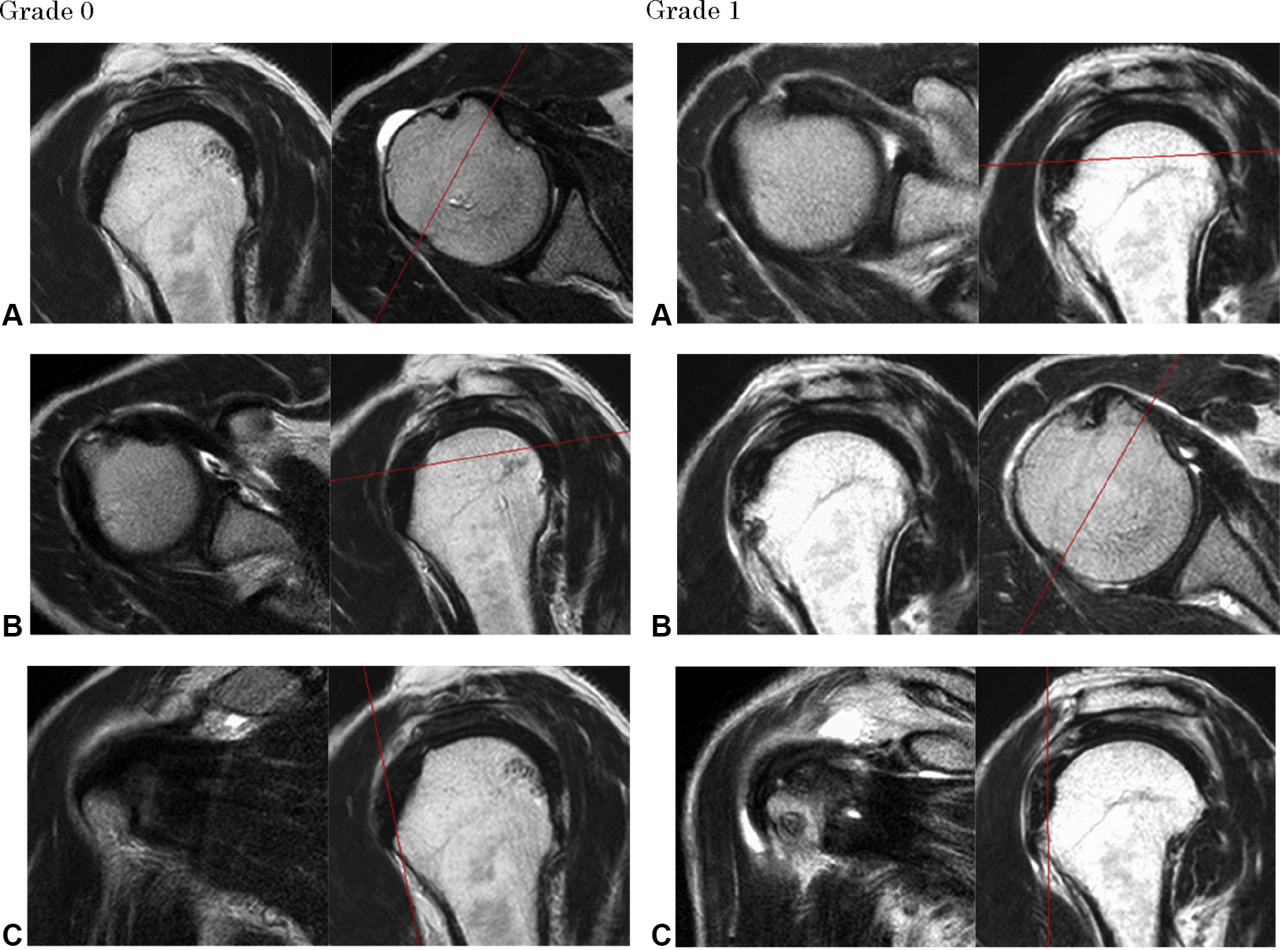

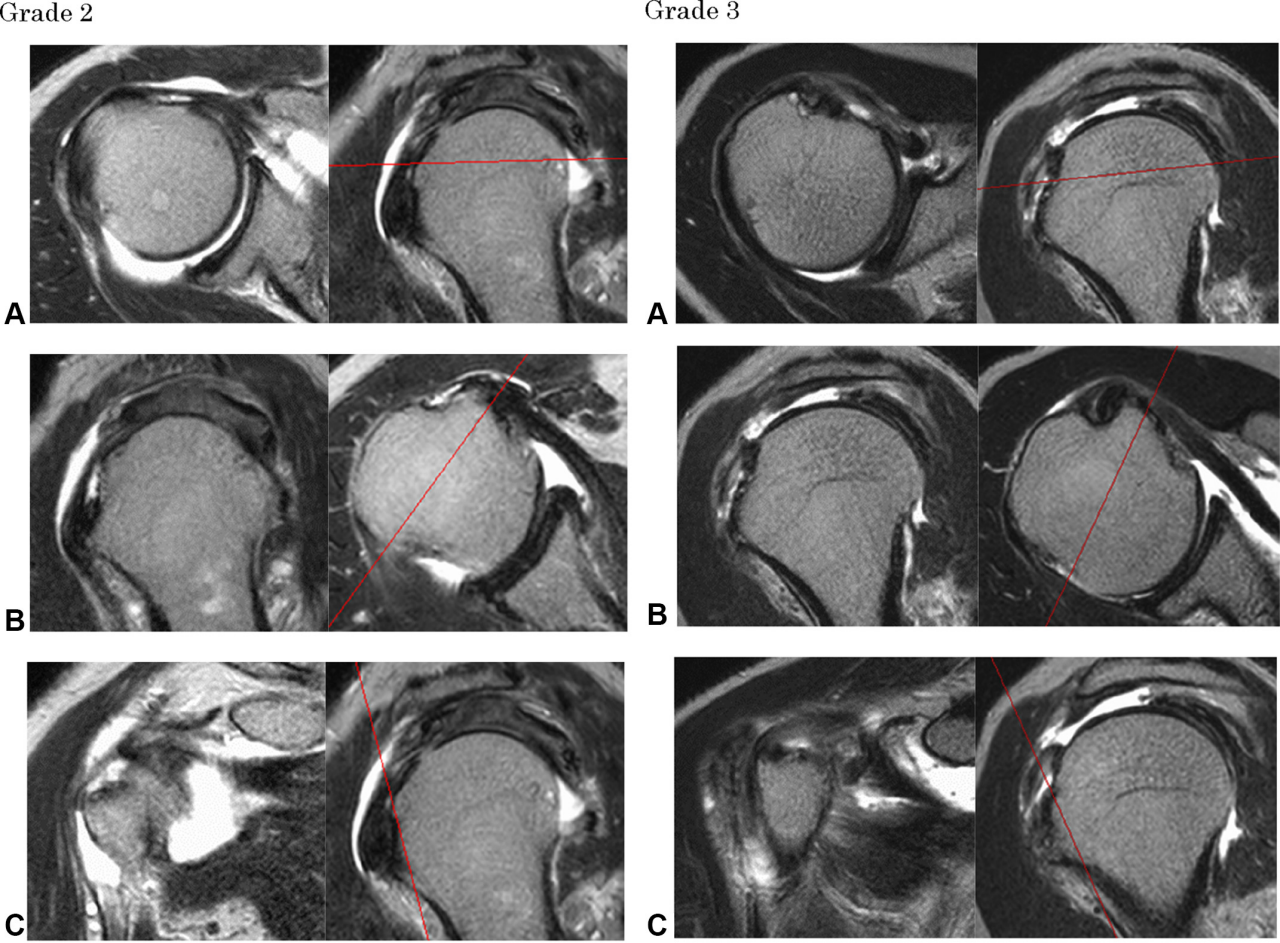
cMRI images of the classification of the anterosuperior aspect STT. Grade 0, intact; grade 1, slight thinning or high intensity of the SSC; grade 2, considerable thinning of the SSC; and grade 3, complete tear. (**A**) Axial view of cMRI; (**B**) Oblique sagittal view of cMRI; (**C**) Oblique coronal view of cMRI. *cMRI*, conventional magnetic resonance imaging; *STT*, subscapularis tendon tear; *SSC*, subscapularis tendon.

All MRI images were blindly and independently checked by an orthopedic surgeon who had more than 4 years of experience and no information about the clinical data. First, we assessed the anterosuperior aspect of the SSC by combining axial, oblique coronal, and oblique sagittal views of cMRI on T2-weighted and fat-suppression (T2FS) images. We classified those images into four grades as follows: grade 0, intact; grade 1, slight thinning or high intensity of the SSC; grade 2, considerable thinning of the SSC; and grade 3, complete tear (Fig. 2). If there was a single image of grade 2 or 3, it was considered as STT.

**Figure 3:**
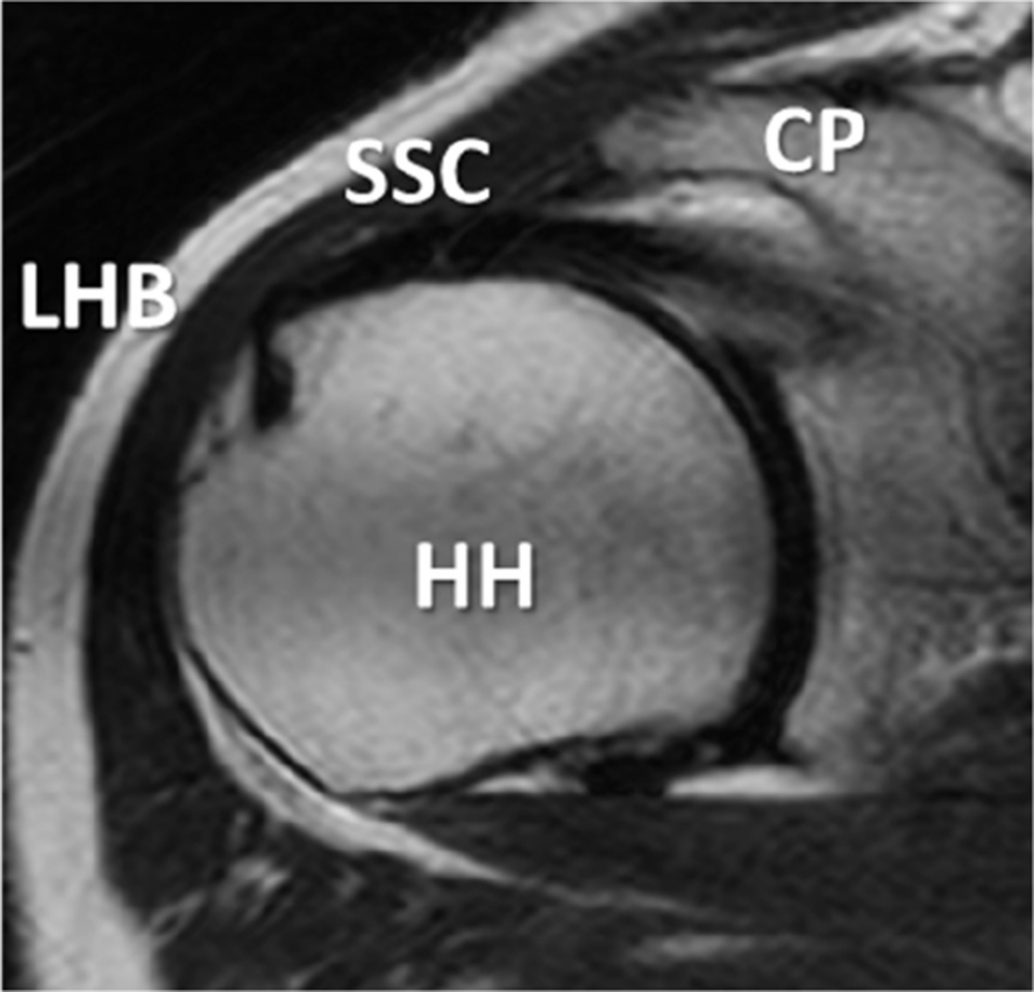
Slice level at which STT status is assessed. *CP*, coracoid process; *HH*, humeral head; *LHB*, long head of biceps brachii; *SSC*, subscapularis tendon; *STT*, subscapularis tendon tear.

**Figure 4:**
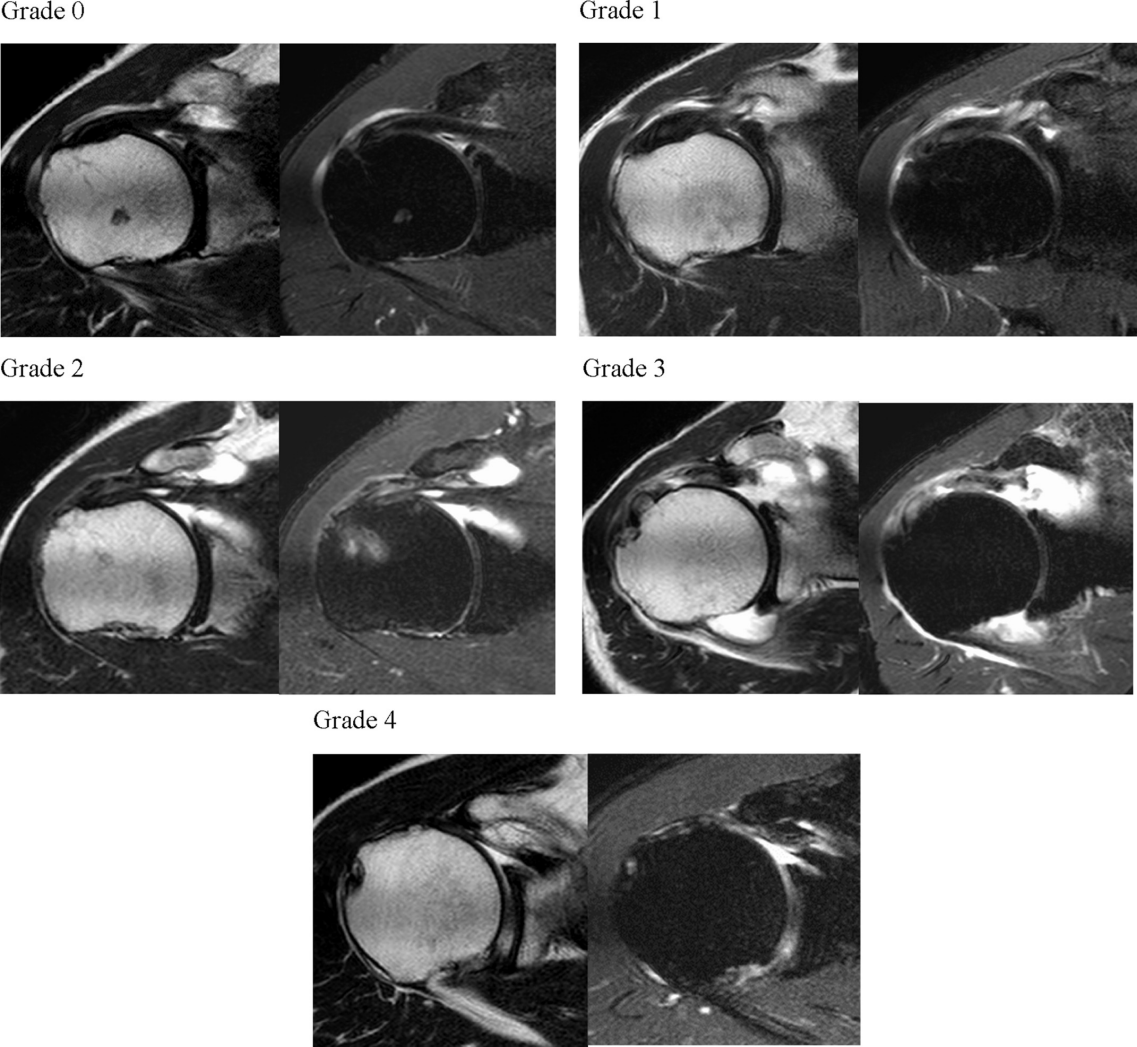
rMRI images of the classification of the anterosuperior aspect STT. Grade 0, intact; Grade 1, slight thinning of the SSC; Grade 2, considerably thinning of SSC; Grade 3, complete tear; Grade 4, coracoid process contact and anterior potion of humeral head. The *Left* images are T2-weighted, and the *Right* images are T2FS. *rMRI*, radial-sequence MRI; *STT*, subscapularis tendon tear; *SSC*, subscapularis tendon.

Then, for rMRI evaluation, we used T2-weighted and T2FS images on a few slices around the base of the coracoid process (Fig. 3). Those were classified into five grades: grade 0, intact; grade 1, slight thinning or high intensity of the SSC; grade 2, considerable thinning of the SSC; grade 3, complete tear; and grade 4, contact of the coracoid process and anterior portion of the humeral head (Fig. 4). We considered grades 0 and 1 of our classification as no tear.

### Arthroscopic findings

All arthroscopic procedures were performed by a senior surgeon with more than 15 years of experience. Surgery was performed with general anesthesia only, or general anesthesia with interscalene block, in the beach chair position. For the first observation, a standard posterior portal was used to assess the SSC within the glenohumeral joint. We created additional portals depending on the condition of the rotator cuff or surgical plan. We also carefully confirmed the SSC insertion attached to the SSC from the subacromial bursa. With regards to arthroscopic findings, Lafosse’s classification was used for assessment of the SSC as follows: intact; type 1, partial lesion of the superior one-third; type 2, complete lesion of the superior one-third; type 3, complete lesion of the superior two-thirds; and type 4, complete lesion of the tendon. We did not treat intact and Lafosse’s classification type 1 lesions because we did not repair or débride in such cases.^18^ We regarded Lafosse’s classification type 2-4 as STT and treated the lesions.

### Interobserver and intraobserver reliability

To investigate intraobserver reliability, the same orthopedic surgeon re-evaluated all MRI images at intervals of more than one month from the initial evaluation. To evaluate interobserver reliability, another orthopedic surgeon with five years of general orthopedic experience similarly evaluated randomly selected cases.

### Statistical analysis

The MRI and arthroscopic findings were collected and compared, and the sensitivity, specificity, PPV, and NPV for cMRI and rMRI were calculated. Statistical analyses to assess the accuracy of cMRI and rMRI were performed. Significance was set at *P* < .05. Interobserver and intraobserver reliabilities were analyzed using the kappa statistic (κ) defined as follows: κ < 0.4 = poor; 0.4 < κ < 0.6 = moderate; 0.6 < κ < 0.8 = good; 0.8 < κ = excellent.^17^ All statistical analyses were performed using IBM SPSS Statistics for Windows, version 20 (IBM Corp., Armonk, NY, USA).

## Results

The results for intraobserver and interobserver reliability are shown in Table I. The intraobserver and interobserver reliabilities for the rMRI classification were κ = 0.78 and 0.65, respectively, corresponding to high reproducibility, and defined as good. Upon assessing whether STT was present or not using rMRI, the intraobserver and interobserver reliabilities were κ = 0.92 and 0.87, respectively, corresponding to high reproducibility, and defined as excellent.

**Table I:** Intraobserver and interobserver reliability of our classification

Reliability	Classification (κ)	STT or not (κ)
Intraobserver reliability	0.78	0.92
Interobserver reliability	0.65	0.87

*STT*, subscapularis tendon tear; *MRI*, magnetic resonance imaging; *rMRI*, radial-sequence MRI.

Intraoperatively, 53 shoulders had STT among 196 shoulders, with a rate of 27.0%. We found 84 patients with intact tendons (42.9%), 59 patients with Lafosse type 1 (30.1%), 38 patients with type 2 (19.4%), 11 patients with type 3 (5.6%), and four patients with type 4 (2.0%).

In preoperative cMRI findings, 166 patients had no tear and 30 patients had STT. The sensitivity of STT was 46.3%, specificity was 96.5%, accuracy was 82.7%, PPV was 83.3%, and NPV was 82.5%.

On preoperative rMRI findings, 49 patients were grade 0 (25.0%), 81 patients were grade 1 (41.3%), 48 patients were grade 2 (24.5%), 16 patients were grade 3 (8.2%), and two patients were grade 4 (1.0%). The sensitivity of STT was 92.5%, specificity was 88.1%, accuracy was 89.3%, PPV was 74.2%, and NPV was 96.9% (Table II). The sensitivity (*P* < .001), accuracy (*P* = .04), and NPV (*P* < .01) in rMRI were significantly higher than those in cMRI. The specificity in cMRI was significantly higher than that in rMRI (*P* = .02). The accuracy of cMRI related to Lafosse’s classification was 97.6% for intact, 93.2% for type 1, 34.2% for type 2, 81.8% for type 3, and 75.0% for type 4 (Table III). The accuracy of rMRI related to Lafosse’s classification was 97.6% for intact, 74.6% for type 1, 89.5% for type 2, 100.0% for type 3, and 100.0% for type 4 (Table IV).

**Table II:** MRI evaluation of STT using cMRI and rMRI.

MRI	Sensitivity	Specificity	Accuracy	PPV	NPV
cMRI	46.3	96.5	82.7	83.3	82.5
rMRI	92.5	88.1	89.3	74.2	96.9

*MRI*, magnetic resonance imaging; *STT*, subscapularis tendon tear; *rMRI*, radial-sequence MRI; *cMRI*, conventional MRI; *PPV*, positive predictive value; *NPV*, negative predictive value.

**Table III:** Accuracy of cMRI related to Lafosse’s classification.

Lafosse’s classification	Intact	Type 1	Type 2	Type 3	Type 4
cMRI STT-	82	55	25	2	1
cMRI STT+	2	4	13	9	3
Accuracy	97.6	93.2	34.2	81.8	75.0

*STT*, subscapularis tendon tear; *cMRI*, conventional magnetic resonance imaging.

**Table IV:** Accuracy of rMRI related to Lafosse’s classification.

Lafosse’s classification	Intact	Type 1	Type 2	Type 3	Type 4
rMRI STT-	82	44	4	0	0
rMRI STT+	2	15	34	11	4
Accuracy	97.6	74.6	89.5	100.0	100.0

*STT*, subscapularis tendon tear; *rMRI*, radial-sequence magnetic resonance imaging.

## Discussion

In this study, we evaluated STT by 1.5 T rMRI using a newly created classification. STT was prevalent, with a sensitivity of 92.5%, specificity of 88.1%, accuracy of 89.3%, PPV of 74.2%, and NPV of 96.9%. Our study provides evidence that rMRI is an effective tool to evaluate STT, especially before surgery.

MRI has been widely used to diagnose RCTs. For diagnosis using MRI, it is possible to diagnose RCT more accurately by evaluating various types of images, such as axial, oblique sagittal, oblique coronal, T1-weighted image, T2-weighted image, and fat suppression. However, with cMRI, it was reported that the sensitivity of STT was low, ranging from 36% to 78%.^1^^,^^2^^,^^7^^,^^20^^,^^21^ This low sensitivity is because the anterosuperior aspect of the SSC is described obliquely in axial, oblique coronal, and oblique sagittal views and becomes unclear owing to the partial volume effect.^16^ Thus, it is difficult to evaluate the anterosuperior aspect of the SSC using cMRI. On the other hand, a study on the diagnosis of STT of the anterosuperior aspect by cMRI reported that the sensitivity of a superior one-third tear was 67%, a superior two-third tear was 82%, and a complete tear was 100%.^22^ It is especially difficult to diagnose a superior one-third tear. In the sagittal oblique view of magnetic resonance arthrography, good results were reported in which the sensitivity was 73%, the specificity was 83%, and the accuracy was 79%.^26^ However, magnetic resonance arthrography is an invasive examination as it requires an injection into the shoulder joint.

rMRI is reported as a useful technique to evaluate the condition of the acetabular labrum.^12^^,^^13^^,^^16^ We consider that rMRI adapted to the shoulder may be a more powerful diagnostic tool than cMRI to accurately diagnose anterosuperior aspect tears. The rotator cuff tendons are also attached to the humeral head in a circumferential shape, similar to the acetabular labrum. Because rMRI draws radially from the center of the humeral head, any part of the rotator cuff tendons can be described vertically. Therefore, the anterosuperior aspect of the SSC may be described more clearly and evaluated more accurately. Some studies have been performed using rMRI for the shoulder.^8^^,^^14^ The imaging technique for rMRI is the same as that for cMRI, except for the slice setting. Filming this radial-sequence image takes approximately 5 min, and it is almost the same as oblique coronal images and axial images of cMRI. All the tendon insertions of the rotator cuff can be assessed with one sequence by using rMRI. Therefore, rMRI provides wider visualization for the rotator cuff with shorter imaging time than cMRI.^14^

Furukawa et al^8^ investigated 55 patients who underwent arthroscopic rotator cuff repair for RCT and compared the diagnostic accuracy of cMRI and rMRI using 3.0 T MRI. They reported good results—sensitivity of STT was 94.7%, specificity was 82.4%, and accuracy was 90.9%—because the rMRI can capture a clear image of the anterosuperior aspect of the subscapularis in the vertical direction. However, that study had a small cohort and used 3.0 T MRI. There is no study of rMRI using only 1.5 T MRI. 3.0 T MRI is also superior to 1.5 T MRI in terms of diagnostic performance;^4^^,^^11^ however, it is more expensive and not popular. It may be difficult to diagnosis STT using rMRI if the user is unfamiliar with the system, and there has also been no classification for the insertion of STT on rMRI. In this study, we had a larger number of subjects and attained a favorable outcome comparatively using 1.5 T rMRI only. Our study provides evidence that the classification made the rMRI findings more intelligible, and we can accurately diagnose using 1.5 T rMRI. The strength of this study is that we could evaluate the insertion of STT and obtain better results using 1.5 T MRI, which is more cost-effective and is often distributed instead of 3.0 T MRI, as reported by Furukawa et al.^8^

There are many studies related to the evaluation of STT. For evaluation by physical examination, Gerber and Krushell^10^ described the lift-off test for examination of an isolated STT. It was reported in a study that the sensitivity was 35%, specificity was 98%, PPV was 90%, and NPV was 76%.^15^ Gerber et al^9^ also reported a belly press test, where sensitivity was 34%, specificity was 96%, PPV was 79%, and NPV was 78%.^15^ These methods are useful as evaluations that can be performed easily and noninvasively, but all have low sensitivity and are widely used as auxiliary tools for diagnosis.

The LHB passes through the bicipital groove just lateral to the SSC attachment area, and the condition of the insertion of the SSC relies on the stability of the LHB.^3^ Because STTs originate from the anterior upper part of the shoulder,^2^^,^^27^ it is thought that LHB and pully lesions often occur concurrently.^9^^,^^28^^,^^30^^,^^32^ STT alone may be asymptomatic, but anterior shoulder pain may be associated with LHB and pulley lesions. Therefore, an accurate assessment of STT and peripheral structures is important.

Recently, ultrasonic diagnostic methods have become a popular approach to evaluate RCT. Furthermore, the dynamic evaluation of ultrasonography improves diagnostic accuracy, and special assessments can be performed, which are impossible in static images. Narasimhan et al^24^ reported that sensitivity was 39.5%, specificity was 93.1%, and accuracy was 75.8% by ultrasonographic evaluation. The sensitivity was low, as for physical examinations, and diagnosing RCT using ultrasonography is not plausible. The shoulder is technically difficult to evaluate with sonography because of the curved nature of many shoulder structures, thus increasing susceptibility to anisotropic artifacts. The diagnostic ability of ultrasound depends on the operator and requires a long time to master the technique.^6^ Ultrasonography cannot describe the area under the bone structures, such as the acromion and coracoid process, because of posterior acoustic shadowing.^29^ Moreover, MRI has some advantages over ultrasonography. MRI can evaluate deeper areas, bone lesions, and muscle quality, which are difficult to describe using ultrasonography. However, the accuracy of diagnosis may increase as technology develops. With the increasing number of orthopedic surgeons who can use ultrasonography, it may become a useful diagnostic tool in the future.

This study had some limitations. First, this study was conducted retrospectively, so there was inherent observer bias; prospective studies are needed to achieve higher levels of evidence. Second, this study had the problem of selection bias because we only included patients who underwent surgery. Third, MRI protocol in this study included thicker slices than is standard. This may affect these results. Fourth, the accuracy of rMRI related to Lafosse’s classification type 1 was low because the insertion of the SSC appeared more excessive in the T2SF image. Therefore, false positives may have increased, but this may be investigated more carefully by arthroscopy. Finally, we did not assess the LHB and pully lesions. We think that STT evaluation including the LHB and pully lesions will be necessary in the future. To do that, 3.0 T MRI may be better than 1.5 T MRI.

## Conclusion

In this study, we evaluated STT by 1.5 T rMRI using a newly created classification. STT was prevalent, with a sensitivity of 92.5%, specificity of 88.1%, accuracy of 89.3%, PPV of 74.2%, and NPV of 96.9%. Our study provides evidence that rMRI is an effective tool to evaluate STT.

## Disclaimers

Funding: No funding was disclosed by the authors.

Conflicts of interest: The authors, their immediate families, and any research foundation with which they are affiliated have not received any financial payments or other benefits from any commercial entity related to the subject of this article.
